# Primary Renal Lymphoma: Long-Term Results of Two Patients Treated with a Chemotherapy + Rituximab Protocol

**DOI:** 10.1155/2012/726424

**Published:** 2012-09-11

**Authors:** F. Vázquez-Alonso, I. Puche-Sanz, C. Sánchez-Ramos, J. Flores-Martín, J. Vicente-Prados, J. M. Cózar-Olmo

**Affiliations:** ^1^Department of Urology, Hospital Universitario Virgen de las Nieves, 18014 Granada, Spain; ^2^Department of Hematology, Hospital Universitario Virgen de las Nieves, 18014 Granada, Spain

## Abstract

Primary renal lymphoma (PRL) is a rare disease of which the etiology and pathogenesis remain controversial, and there is currently no standard treatment for it. We present the results of a long-term followup of two patients who were diagnosed with PRL and treated with cyclophosphamide, adriamycin, vincristine, prednisolone and rituximab (CHOP + R) regimen. Both patients reached a complete response, and there is no evidence of recurrence after 4.5- and 5-year followup periods. Based on our experience and other recently published studies, we recommend the combination of CHOP + rituximab as the elective treatment for this disease. To our knowledge, this is the longest followup period with a complete response that has been reported with this modality of treatment.

## 1. Introduction

Lymphomas are malignant tumors that are caused by lymphoid cell proliferation. Involvement of the kidney by lymphoma is a common late manifestation of advanced nodal disease. However, in 1980, Coggins reported the first patient diagnosed with a PRL [1]. Since then, approximately 70 cases of PRL have been reported in the medical literature. Nevertheless, a subsequent paper [2] has demonstrated that less than half of those cases were conformed to the Malbrain's criteria to be considered PRL [3]. PRL accounts for 0.7% of all extranodal lymphomas and represents less than 1% of all renal lesions.

In this paper, we review the status of PRL and its treatment, and we describe the long-term follow-up evaluation of two patients who were treated with a chemotherapy + rituximab protocol.

**Case 1**

The case 1 subject is a 77-year-old woman with a previous history of arterial hypertension. The patient was referred to our department complaining of anorexia, weakness, and having generally felt poorly in the previous months. Slight renal function impairment appeared in the blood tests (i.e., serum creatinine = 1.61 mg/dL and urea = 64 mg/dL). Ultrasonography revealed a left solid renal mass with a compressed left renal vein. An MRI confirmed a 9.6 × 8.1 × 8.8 cm polylobulated renal mass that had invaded the renal vein and was surrounded by multiple adenopathies. Suspecting renal cell carcinoma, the patient underwent a left radical nephrectomy. However, the pathologic study revealed a diffuse large B-cell lymphoma (CD20+, BCL-2+, CD10+, BCL-6−, CD30−, p53−, and Ki67 90%) with extended necrotic areas that had infiltrated the perirenal fat and hilar lymphatic nodes. A CT scan demonstrated that the disease had disseminated to the lungs. A bone marrow biopsy in the iliac crest was performed and exhibited no neoplastic infiltration. The patient was treated with six cycles of CVP + R. No significant adverse effects were observed, and a complete response of the disease was reached. After a 5.5-year follow-up period, the patient is asymptomatic and presents no evidence of recurrence.

**Case 2**

**Figure 1:**
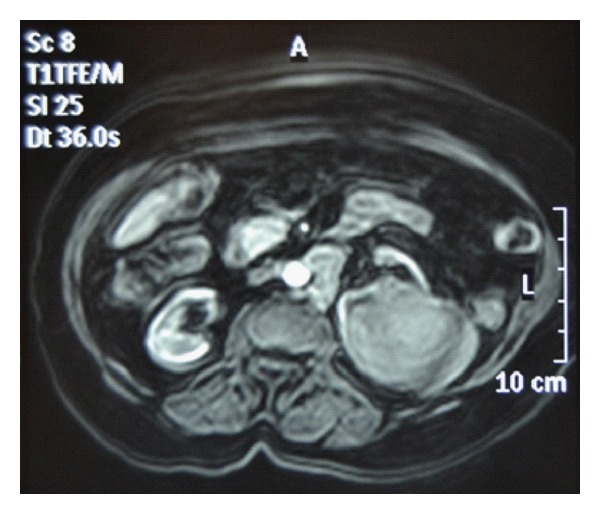
In the MRI, PRL is differentiated from RCC based on the lower signal intensity on unenhanced T1-weighed images compared with a normal renal cortex and fewer enhancements on early gadolinium-enhanced images.

**Figure 2:**
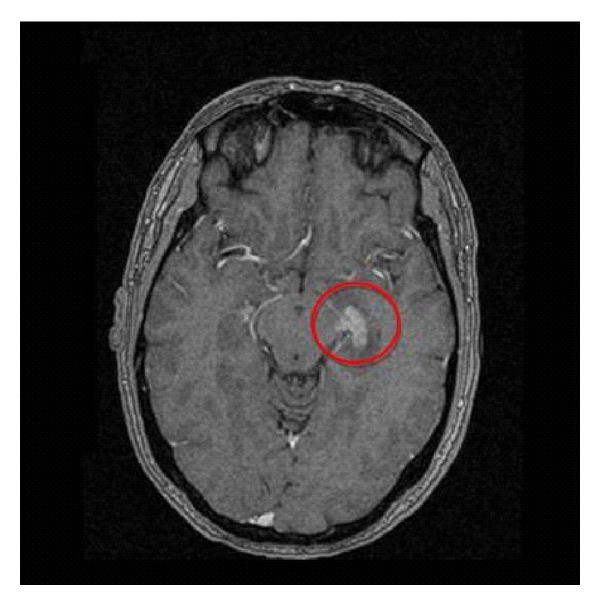
PET scan revealed a well-delimited lesion in the left temporal region that suggested metastasis.

The case 2 subject is a 46-year-old man with no previous history of interest. In this case, the patient presented with weight loss and left flank pain during the 6 previous months. Blood tests showed renal function impairment (i.e., serum creatinine = 1.80 mg/dL and urea = 95 mg/dL). A CT scan revealed an 11cm irregular mass on the left kidney, with hypodense areas that suggested necrosis. No urinary tract obstruction was observed. The study was completed with an MRI (Figure 1). Suspecting renal lymphoma, a percutaneous biopsy was performed. The histology confirmed a diffuse large B-cell lymphoma (CD45+, CD20+, BCL+, BCL6+/−, CD10−, ALK−, CD30−, p53+ in 30% of the cells, and Ki67 80%). The bone gammagraphy and the bone marrow biopsy were negative. The patient was treated with six cycles of CHOP + R. The treatment tolerance was good. A follow-up CT scan indicated a complete response of the renal lesion. One year later, the patient was admitted for convulsions associated with olfactory hallucinations and headache. PET and cranial MRI scans revealed a well-delimited lesion in the left temporal region that suggested metastasis (Figure 2). The course of action was decided to be stereotactic radiosurgery of this lesion. A complete response was achieved. After a 5-year follow-up period, the patient is asymptomatic and has no evidence of recurrence.

## 2. Discussion

The term PRL is attributed when the disease is localized to the kidney without any sign of other organ involvement or in which renal involvement is the presenting manifestation [4]. PRL is a rare and uncertain entity because renal parenchyma lacks lymphatic tissue. Therefore, the status of PRL as a primary disease or the first manifestation of a rapidly progressive systemic disease is controversial. Malbrain proposed several criteria to diagnose PRL as follows: (i) acute renal failure at the presentation in the absence of other causes of renal impairment; (ii) rapid improvement of renal function after treatment; (iii) increased kidney size without any urinary tract obstruction; (iv) an absence of other nodal involvement beyond the kidney; (v) a confirmed diagnosis made by biopsy [3]. 

PRL usually appears in males in their sixties. Renal involvement is normally unilateral; an affection of both sides is unusual. The clinical presentation is similar to the other renal malignancies. Flank or abdominal pain is the most frequent symptom. However, PRL can present with proteinuria or nephrotic syndrome and rapidly progress to renal failure, especially when both kidneys are affected. PRL has been associated with inflammatory and infectious chronic diseases, such as chronic pyelonephritis, Sjögren's syndrome, systemic erythematous lupus, or Epstein-Barr virus [2]. 

LRP is often mistaken for a RCC, and the diagnosis is performed after a radical nephrectomy. However, imaging studies may provide evidence for suspected cases of PRL. Ultrasonographic images typically depict an unspecific homogeneous hypoechoic mass. Therefore, a CT scan is preferred to differentiate PRL from an RCC. PRL usually appears as a hypervascularized mass with minimal and characteristic homogeneous contrast enhancement. Other indirect signs of LRP include the following: renal size enlargement, sinus or hilum direct infiltration by a bulky mass, or diffuse retroperitoneal infiltration. In contrast, indirect signs that may indicate an RCC include the following: the presence of calcifications, venous thrombosis, or an obstructive mass effect over the renal vessels or urinary tract. However, a percutaneous biopsy is always required to confirm the diagnosis. Bone gammagraphy and a bone marrow biopsy should always be performed to exclude extrarenal dissemination.

Diffuse large B-cell lymphoma (DLBCL) is the most common histology of PRL. Recent research has focused on intravascular large B-cell lymphoma (IVLBCL), which is a rare variant of DLBCL that is characterized by malignant lymphoid cell proliferation within the lumina of small blood vessels of several organs (i.e., central nervous system, kidneys, adrenals, skin, liver, or lungs). The presentation of IVLBCL that is limited to the kidney is rare. However, unlike the fatal prognosis of systemic IVLBCL, it was observed that patients having kidney-limited IVLBCL had a good prognosis when treated with CHOP + rituximab protocols. Therefore, some authors recommend differentiating kidney-limited IVLBCL as a distinct, better-prognosis variant of systemic IVLBCL [5, 6]. It remains unknown whether renal IVLBCL is a subtype of PRL or simply the first manifestation of systemic IVLBCL. 

Systemic chemotherapy is currently the first treatment option for PRL. Although most authors believe that the CHOP protocol should be an elective option (as it is in non-Hodgkin's BCL), there is no agreed-upon standard treatment approach for PRL. In patients with cardiopathy, adriamycin (H) should be avoided. For this reason, we chose the alternative CVP regimen to treat the case 1 subject. Evidence supporting the efficacy of a combined CHOP + rituximab protocol compared with CHOP alone in the treatment of DLBCL has been well documented. A recent retrospective study compared both regimens in treatment of systemic IVLBCL and demonstrated that PFS and OS rates at 2 years after diagnosis were significantly higher for patients in the chemotherapy + rituximab group (PFS, 56%; OS, 66%) compared with patients in the chemotherapy group (PFS, 27%; OS, 46%) [7]. 

Rituximab is a chimeric monoclonal antibody against the CD20 antigen, which is expressed by normal pre-B and mature B lymphocytes. However, the physiopathological mechanisms that explain the mode of action of rituximab are not well known. There is accumulating preclinical evidence that B cells can downregulate T-cell-mediated antitumor immunity. Nevertheless, this hypothetical immune-booster mechanism of action that rituximab could achieve by depleting the B-cell count has not been clinically confirmed in humans. The experience with rituximab in neoplastic patients did demonstrate a rapid decrease of circulating B cells, but the absence of significant changes in other immunological parameters suggested an incomplete depletion of the whole B-cell population. According to this contradiction, an exhaustive review of the clinical uses of rituximab has indicated that rituximab's mechanism of action might vary from one patient to another as well as from one disease to another. Thus, B cells could not be the only target of rituximab, and T cells could be indirectly and differently modulated [8].

Rituximab has a good safety profile, and important adverse effects are rare. However, the long-term effects of B-cell depletion and hypogammaglobulinemia associated to repeated cycles are unknown. To our knowledge, and although in case 2 further radiosurgery was needed, this is the longest follow-up period with a complete response reported to date with this modality of treatment, and no relevant adverse effects are reported.

PRL has a poor prognosis with rapid dissemination and a 75% mortality rate one year after diagnosis. However, our case subject experiences were different. Therefore, we hypothesize that early diagnosis combined with chemotherapy + rituximab could improve survival rates. Strict follow-up is essential to determine the recurrence rate over time.

## 3. Conclusion

PRL is a rare disease in which the etiology and pathogenesis are unclear. There is no standardized treatment for PRL. In the absence of clinical trials due to a shortage of cases, according to our experience and other recently published studies [5, 6, 9, 10], we recommend the administration of CHOP + rituximab as the elective treatment for this disease.
